# A Survey on Saffron in Major Islamic Traditional Medicine Books

**Published:** 2013-01

**Authors:** Behjat Javadi, Amirhossein Sahebkar, Seyed Ahmad Emami

**Affiliations:** 1 *Department of Traditional Pharmacy, Faculty of Traditional Medicine, Tehran University of Medical Sciences, Tehran, Iran*; 2 *Biotechnology Research Center and School of Pharmacy, Mashhad University of Medical Sciences, Mashhad, Iran*; 3 *Department of Pharmacognosy, School of Pharmacy, Mashhad University of Medical Sciences, Mashhad, Iran*

**Keywords:** Crocus sativus, Saffron, Traditional medicine

## Abstract

Islamic Traditional Medicine (ITM) is a holistic system of medicine. Saffron (*Crocus sativus*) is one of the most famous plants cultivated in Iran and has a wide range of activities such as oxytocic, anti-carcinogenic, exhilarant, anti-depressant, and anti-asthma effects. In addition, saffron can increase the bioavailability and enhance absorption of other drugs. This study comprises a bibliographical survey of 13 major ITM books regarding different medical aspects of this species. Ferdows al-Hekmah fi’l-Tibb (The Paradise of Wisdom in Medicine), Al-Hawi fi’l-Tibb (Comprehensive Book of Medicine), Kamel al-Sanaat al-Tibbyyah (Complete Book of the Medical Art), Al-Qanun fi’l-Tibb (Canon of Medicine), Zakhireh Kharazmshahi (Treasure of Kharazmshahi), and Makhzan al-Adwiah (Drug Treasure) are some of the most important ITM books used in this survey.

## Introduction

Islamic Traditional Medicine (ITM) is a holistic system of medicine which dates back to 14 centuries ago. In the history of medicine, ITM or Arabic medicine refers to medicine developed in the medieval Islamic civilization and mostly written in Arabic, the lingua franca of the Islamic civilization. Despite this fact, a significant number of scientists during this period were not Arab. Therefore, the label "Arabic medicine" does not describe the rich diversity of Eastern scholars who have contributed to Islamic science in this era. After the decadence of Greco-Roman medicine, Islamic medicine took over the lead for the following thousand years. Muslims searched for old medical books, read, translated, distributed, and worked upon them (1). The most distinguished and eminent physicians in Islamic territories in the medieval era were Tabari, Razi, Ahwazi, and Ibn Sina. 

ITM is based on the theory of temperament. Temperament is a quality resulting from the interaction of opposite properties present in elements consisting of minute particles. Thus, a uniform quality occurs which is present in all of them. Hotness, coldness, moistness, and dryness are four temperaments that naturally occur in every existing substance including living creatures (2).

Saffron (*Crocus sativus *L*.*) is a species belonging to the Iridaceae family and has been widely used as an herbal medicine, spice, food coloring, and a flavoring agent since ancient times. It is a perennial bulbous plant that grows 8 to 30 cm high. The plant has a large squat tuber surrounded by reticulate and fibrous sheaths. The leaves are erect or splayed, narrow, and have a ciliate margin and keel. The lily-like flowers have two bracts at the base. There is a pale violet-veined calyx, yellow anthers, and white filament. The thread-like style of the plant is 10 mm long and stigma is bright orange (3). This plant is cultivated in Europe, Turkey, Iran, Central Asia, India, China, and Algeria. In Iran, it is cultivated in the south Khorasan province from ancient times (4). The dried stigma and tops of styles constitute the saffron of commerce. 

Crocin, crocetin, and safranal are the main chemical constituents of saffron. The color of saffron is due to the presence of crocins, which have glycoside carotenoid structure. The bitter taste of saffron is attributed to picrocrocin. Safranal is an aromatic aldehyde which is the main component of plant volatile oil (5).

The present essay represents a bibliographical survey of major ITM books in order to summarize the mentioned medicinal uses of saffron, its temperament, adverse effects, and lethal dosage. In addition, the conformity of traditional applications with the findings from modern pharmacological research has been discussed.

***Literature search***

Data on the medicinal uses of saffron were obtained from 13 major books of ITM which were selected from almost 600 accessible books. The selected books (Table 1) were the most important sources of medical science and Materia Medica for centuries. These works were searched for information regarding temperament, general and therapeutic uses, and undesirable effects of saffron. 

## Temperament

In all of the studied books saffron’s temperament is mentioned as warm and dry. As indicated in Table 2, most of the texts introduced saffron as an astringent (qabez), resolvent (mohallel), and concoctive (monzedj) drug. In ITM, these three general effects together with bitterness are responsible for most of other medicinal activities of saffron. 

## Medicinal properties

***Gastro-hepatoprotective effects***

This plant is a powerful liver tonic and hepatic deobstruent. Tabari has described hepatoprotective effects of saffron as: "It is warm, moderate, and dry. It is resolvent and bitter. Therefore, it can treat liver obstructions" (6, 7). Saffron is a gastric tonic and suppresses the appetite. Razi has written: "Saffron is a digestive drug with astringent properties. It cleanses the stomach (8). 

**Table 1:** Information regarding 13 major ITM books that described medicinal effects of saffron

**Author** **Living period**	**Book**	**Language**	**Year and place of publication**
Ali Ibn Rabban Tabari 773-861 A.D.	Ferdows al-Hekmah fi’l-Tibb (The Paradise of Wisdom in Medicine)	Arabic	1928, Berlin
Mohammad Ibn Zakariya Razi (Rhazes) 865-925 A.D.	Al-Hawi fi’l-Tibb (Comprehensive Book of Medicine)	Arabic	1968, Hyderabad
Abu Bakr Akhawayni Bukhari 10^th^ century	Hedayat al-Mota’allemin fi’l-Tibb (An Educational Guide for Medical Students)	Persian	1992, Mashhad
Movaffaq a,ddin Abu Mansur Heravi 10^th^ century	Al-Abniyah an Haqayeq al-Adwiyah (Basics of Realities on drugs)	Arabic	1967, Tehran
Ali Ibn Abbas Majusi Ahwazi (Haly Abbas) 930- 994 A.D.	Kamel al-Sanaat al-Tibbyyah (Complete Book of the Medical Art)	Persian	1877, Bulaq
Hossein Ibn Ali Ibn Sina (Avicenna) 980-1037 A.D.	Al-Qanun fi’l-Tibb (Canon of Medicine)	Arabic	1987, New Delhi
Sayyed Esma’il Jorjani 1042-1136 A.D.	Zakhireh Kharazmshahi (Treasure of Kharazmshah)	Persian	1976, Tehran
ibid	Al-Aghraz al-Tibbyyah val Mabaheth al Alaiiah (Medical Gouls and Allaii’s Discussion)	Persian	1966, Tehran
Zia al-Din Ibn Beytar (Greatest Botanist and Pharmacist of the world of Islam) 1193-1248 A.D.	Al-Jamee Le-Mofradaat al- Adwiah val- Aghziyah (Comprehensive book in Simple Drugs and Foods)	Arabic	2001, Beirut
Ibn Nafis Qarshi 1210-1288A.D.	Al-Mujaz fi’l-Tibb (A Commentary on Ibn Sina’s Canon)	Arabic	2001, Cairo
Dawoud Antaki 1599 A.D.	Tazkereh Oulol-Albab( Memorandum Book )	Arabic	2000, Beirut
Hakim Mohammad Momen Tonekaboni 16^th^ century	Tohfat al-Momenin (Rarity of the Faithful)	Persian	1959, Tehran
Mohammad Hussein Aqili Khorasani 18^th^ century	Makhzan al-Adwiah (Drug Treasure)	Persian	1992, Tehran
			

***Oxytocic properties***

One of the most important effects of saffron is its potent oxytocic activity which is exerted even after local use. Hence, the plant has traditionally been prescribed to facilitate difficult labors. Razi has a note in this regard: "Ingestion of 6 to 7 grams of saffron induces the labor. I myself prescribed it for many times and the results were always successful" (8). Antaki has written: "It has been experienced that oral use of 3.5 g saffron with rose water and sugar can facilitate delivery. Application of a vaginal suppository prepared by 3.5 g of saffron accelerates labor and delivery of the placenta. It has also contraceptive effects (9). 

***Treatment of urogenital disorders***

This plant has also been reported to be useful for the treatment of female genito-urinary system disorders. Heretofore, a number of surveys have indicated the clinically relevant effects of saffron, at different doses, in the management of premenstrual syndrome, dysmenorrhea, and irregular menstruation (10-13). 

**Table 2:** Temperament, medicinal and adverse effects, and lethal dosage of saffron in ITM major books

General medicinal effects	Therapeutic effects	Adverse effects*	Lethal dosage	References
Bitter Resolvent	Liver deobstruent		-	Tabari, 1928 (6)
Astringent Bitter Concoctive Disinfectant	Anti-inflammatory Aphrodisiac Digestive Diuretic Emetic exhilarant Gastric tonic Hypnotic Improve complexion Internal organs tonic Liver tonic Oxytocic Pleurisy Respiratory relaxant Respiratory tonic Visual improvement	Headache Hypomania Loss of appetite Harmful for brain Nausea	10.5 g	Razi, 1968 (8)
Astringent Concoctive Resolvent	Hypnotic Improve complexion Internal organs tonic Liver deobstruent Liver tonic Vascular deobstruent	Harmful for stomach Headache Head congestion (Head fullness) Yellow skin	-	Heravi, 1967 (34)
	Anti-lithiasis Anti-asthma Conjunctivitis Dropsy Dysentery Eye diseases Gastritis Gastrogenic diarrhea Gout Haemoptysis Hemorrhoids Intestinal excoriation Joints pains Liver diseases Pharyngitis Pharyngitis Rectal collapse Spleen diseases Women genital diseases		-	Akhawayni Bukhari, 1992 (45)
Astringent Attenuant Concoctive Desiccant Diuretic	Inflammations of internal organs Internal organs tonic Liver deobstruent		-	Majusi Ahwazi, 1877 (35)
Astringent Concoctive Disinfectant Diuretic Resolvent	Anti-inflammatory Aphrodisiac Cardiac tonic Deobstruent Emetic Exhilarant Eye diseases Gastric tonic Improve complexion Internal organs tonic Liver tonic Otitis Oxytocic Pleurisy Respiratory tonic Spleen diseases Uterine malignancies Uterine sclerosis	Headache Hypnotic Dizziness Nausea Loss of appetite	10.5 g	Ibn Sina, 1987 (2)
Astringent Resolvent	Exhilarant		-	Jorjani, 1976 (14)
Astringent Concoctive Disinfectant Resolvent	Exhilarant Eye diseases Hypnotic Internal organs tonic Respiratory relaxant	Headache dizziness	-	Jorjani, 1966 (46)
Astringent Bitter Concoctive Diuretic Emollient Potent resolvent	Anti-inflammatory Aphrodisiac Detoxification of alcohol Emetic Exhilarant Eye diseases Gastric tonic Hypnotic Improve complexion Internal organs tonic Liver deobstruent Liver tonic Narcotic Otitis Oxytocic Pleurisy Rectal problems Renal & vesical cleanser Respiratory relaxant Respiratory tonic Uterine diseases Vascular deobstruent Visual improvement	Headache Hypomania Loss of appetite Head fullness Nausea	10.5 g	Ibn Beytar, 2001 (47)
Astringent Concoctive Diuretic Resolvent	Cardiac tonic Deobstruent Hypnotic Improve complexion Oxytocic Visual improvement	Loss of appetite Headache	-	Ibn Nafis Qarshi, 2001 (48)
Astringent Resolvent	Anti- lithiasis Aphrodisiac Arthralgia, gout, and back pains Contraceptive Exhilarant Gastric tonic Haemostatic Liver tonic Oxytocic Palpitation Pharyngitis Pleurisy Stimulant Uterine diseases Visual improvement	Headache Harmful for lungs Loss of appetite	10.5 g	Antaki, 2000 (9)
Concoctive Diuretic Resolvent of **infectious** phlegm	Anti- lithiasis Aphrodisiac Arthralgia Cold headache Erysipelas Exhilarant Eye diseases Gout Haemostatic Hypnotic Improve complexion Induce laughter Internal organs tonic Liver deobstruent Liver tonic Malignancies Otitis Oxytocic Rectal diseases Renal & vesical cleanser Respiratory tonic Spleen deobstruent Uterine diseases Uterus malignancies	Dizziness Harmful for nerves Headache Nausea Stupor Weakness	10.5 g	Tonekaboni, 1959 (31)
Astringent Agglutinant Stimulant Treat Phlegmatic infections	Anti-lithiasis Aphrodisiac Arthralgia Brain deobstruent Detoxification of alcohol Eye diseases Gastric tonic Gout Haemostatic Hypnotic Improve complexion Induce laughter Internal organs tonic Liver deobstruent Liver tonic Malignancies Otitis Oxytocic Pleurisy Potent exhilarant Rectal diseases Renal & vesical cleanser Respiratory tonic Severe headache Spleen deobstruent Urinary retention Uterine diseases Uterus malignancies Visual improvement	Dizziness harmful for kidney Headache Loss of appetite Nausea	10.5 g	Aqili Khorasani, 1992 (30)

Most of the mentioned side effects, including headache, are observed following consumption of high doses of saffron

***Antidepressant properties***

One of the most well known effects of saffron is its exhilarant and anti-depressant activity which leads to the sense of happiness and laughter. Jorjani has stated that: "Saffron is astringent and resolvent and its fragrance can strengthen these two effects. Hence, its action on enlivening the essence of the spirit and inducing happiness is great" (14). Modern scientific evidence has also well supported the beneficial impact of saffron stigma and petal extracts as well as crocin in the treatment of mild to moderate depression. The positive effects of saffron in the improvement of depression symptoms have been confirmed by both animal and clinical data and are comparable to those of standard drugs such as imipramine and fluoxetine (15). 

***Aphrodisiac properties***

Saffron also possesses aphrodisiac properties and hence used to cure impotence. There is experimental and clinical evidence indicating that saffron and its bioactive pigment, crocin, could improve sexual behaviors. The positive effects of saffron include increasing of libido, enhancement of erectile function, and amelioration of semen quality (16-20). 

***Treatment of ocular disorders***

Saffron was used to prepare a special eye formulation called collyrium (Kohl) to treat a range of ophthalmic disorders such as cataract and conjunctivitis and to improve vision. The proposed traditional benefits are well consistent with the findings of modern scientific research. Saffron extract along with crocetin and crocin are effective for the enhancement of retinal blood flow (21), protection against tunicamycin- and H_2_O_2_-induced retinal damage (22), treatment of asthenopia (23), and prevention of age-related macular degeneration (21, 24).

***Treatment of respiratory disorders***

It has been traditionally prescribed to improve respiratory function, asthmatic problems, and as a lung tonic. In this context, a relaxant effect on tracheal smooth muscle has been described for this plant (25). Safranal has been reported as a phytochemical that plays an important role in the observed effects (26). Finally, the bronchodilatory effects of saffron could be attributed to the stimulation of β2-adrenergic and H1 histaminergic receptors (27) while blocking the muscarinic receptors (26). 

***Cardioprotective effects***

Saffron is a heart tonic that has been used to support the cardiovascular functions and treatment of palpitation. Several studies have supported the cardioprotective and anti-atherosclerotic effects of saffron-derived bioactive components, crocin, and crocetin (16, 28, 29). The mechanisms underlying the anti-atherosclerotic effects include anti-hyperlipidemic and insulin sensitizing effects, inhibition of foam cell formation, oxidized low-density lipoprotein (LDL) uptake, aortic intima thickening, lipid absorption, and vascular cell adhesion molecule-1 (VCAM-1) expression, while boosting fecal fat excretion (16). 

***Anti-cancer effects***

The effects of saffron in the treatment of tumors and malignancies, in particular uterus malignancies, have been mentioned in *Canon of Medicine *and some other studied books (2, 30, 31). Ibn Sina has noted that: "Local application of saffron with beeswax or egg yolk and olive oil is effective to treat uterus malignancies" (2). During recent years, there has been a pile of *in-vitro* and *in-vivo* evidence indicating the promising anti-carcinogenic effects of saffron and, in particular, its bioactive phytochemicals (crocin, crocetin, diglucosylcrocetin, and dimethylcrocetin) against different types of cancer. Such broad-spectrum antitumor properties of saffron is deemed to be due to its modulatory effects on gene expression, induction of conformational changes in DNA, induction of apoptosis, modulation of sigma-1 receptors, and scavenging of free radicals and inhibition of topoisomerase II (16, 32, 33).

***Absorption enhancing properties***

In addition to the aforementioned indications, a very special effect has been reported by some of the mentioned authors regarding saffron which is the ability to increase the bioavailability and enhance absorption of other drugs. This action can increase the effects of a potent drug with undesirable effects which cannot be prescribed in high doses (8, 21, 31, 34, 35).

***Anti-inflammatory properties***

Another important biological activity of saffron which has been mentioned in most of the studied books is its anti-inflammatory effects. Anti-inflammatory properties of saffron and crocin have also been approved by recent studies and in different models of inflammation (36-38). Most of these beneficial effects of saffron in the mitigation of inflammation have been attributed to crocin and crocetin. Besides, the observed anti-inflammatory properties have been suggested to the positive impact of saffron and its phytochemicals in the enhancement of antioxidant enzymes as well as scavenging of reactive oxygen species which are key mediators in the promotion of oxidative stress and subsequent inflammatory response (39).

## Toxicity and adverse effects

Findings of *in-vivo* studies have revealed that saffron has negligible toxicity. Oral LD_50_ of saffron decoction in mouse has been reported to be 20.7 g/kg. Higher doses could be lethal due to the toxic effects on central nervous system and kidneys. Oral administration of saffron extract at doses between 0.1-5.0 g/kg has been reported to be non-toxic in mouse model. Clinical data on the toxicity and safety of saffron have been inconsistent. Daily consumption of saffron up to 1.5 g/day has not been found to be associated with any adverse effect. However, doses higher than 5 g are toxic, and at 20 g are lethal. Saffron doses over 10 g have been used for abortion. At this latter dose, saffron can induce vomiting, uterus bleeding, hematuria, gastrointestinal bleeding, and vertigo (40).

The most frequent adverse effects of saffron mentioned in studied books were headache, nausea, head fullness, dizziness, hypomania, and appetite suppression. Regarding the undesirable effects of saffron, Aqili has stated that: "It can cause headache and its consumption with wine results in intoxication. Long-term use of saffron can lead to dizziness and damage to nervous system. Aniseed and oxymel can correct these adverse effects" (30). Skin yellowing is another side effect reported for saffron (34). Modern scientific studies have also implied that colored constituents of saffron may accumulate in sclera, skin, or mucosa, thus mimicking icteric complaints (41). Lethal dosage of saffron has been stated to be about 10.5 g. In Canon, Ibn Sina has stated regarding the lethal dosage of saffron as follows: "Intake of 10.5 g of saffron is fatal due to the induction of extreme joy." This issue has been proved by the literature (3).

## Quality assessment

Ibn Sina introduced high-quality saffron as follows: "Fresh saffron of high quality is characterized by nice color and fragrance. The upper parts of its stigma should be whitish in color. Saffron should not be moldy. It should be neither too compact and thick nor crumbling. Besides, it should not easily impart its color on touch" (2).

## Conclusions

ITM literature research can play an important role in retrieving valuable data regarding medicinal uses of natural products. Traditional uses of saffron have been consistently confirmed by modern pharmacological and clinical investigations (42, 43). The present essay, along with another recent interesting review (44), provides an insight on the importance of bibliographical surveys on ITM books in order to provide medical and pharmacological records of plants with possible bioactive properties.
